# Aldehyde Dehydrogenase 2 Family Member (ALDH2) Is a Therapeutic Index for Oxaliplatin Response on Colorectal Cancer Therapy with Dysfunction p53

**DOI:** 10.1155/2022/1322788

**Published:** 2022-02-07

**Authors:** Wei-Lin Wang, Uyanga Batzorig, Chin-Sheng Hung, Po-Li Wei, Chien-Yu Huang, Yu-Jia Chang

**Affiliations:** ^1^Graduate Institute of Clinical Medicine, College of Medicine, Taipei Medical University, Taipei, Taiwan; ^2^Division of Colorectal Surgery, Department of Surgery, Taipei Medical University Hospital, Taipei Medical University, Taipei, Taiwan; ^3^International PhD Program in Medicine, College of Medicine, Taipei Medical University, Taipei, Taiwan; ^4^Department of Dermatology, University of California, San Diego, La Jolla, CA 92093, USA; ^5^Department of Surgery, School of Medicine, College of Medicine, Taipei Medical University, Taipei, Taiwan; ^6^Cancer Research Center and Translational Laboratory, Taipei Medical University Hospital, Taipei Medical University, Taipei, Taiwan; ^7^Graduate Institute of Cancer Biology and Drug Discovery, Taipei Medical University, Taipei, Taiwan; ^8^Division of General Surgery, Department of Surgery, Shuang Ho Hospital, Taipei Medical University, Taipei, Taiwan; ^9^Division of Colorectal Surgery, Department of Surgery, Taipei Medical University Shuang Ho Hospital, New Taipei City 235041, Taiwan; ^10^Department of Pathology, Wan Fang Hospital, Taipei Medical University, Taipei, Taiwan; ^11^Cell Physiology and Molecular Image Research Center, Wan Fang Hospital, Taipei Medical University, Taipei, Taiwan

## Abstract

Oxaliplatin resistance is a major issue in the treatment of p53 mutant colorectal cancer (CRC). Finding the specific biomarkers would improve therapeutic efficacy of patients with CRC. In order to figure out the biomarker for CRC patients with mutant p53 access oxaliplatin, a Gene Expression Omnibus dataset (GSE42387) was used to determine differentially expressed genes (DEGs). The Search Tool for the Retrieval of Interacting Genes (STRING) and Cytoscape software were used to predict protein-protein interactions. The Database for Annotation, Visualization, and Integrated Discovery online tool was used to group the DEGs into their common pathways. 138 DEGs were identified with 46 upregulated and 92 downregulated. In the PPI networks, 7 of the upregulated genes and 13 of the downregulated genes were identified as hub genes (high degrees). Four hub genes, aldehyde dehydrogenase 2 family member (ALDH2), aldo-keto reductase family 1 member B1 (AKR1B1), aldo-keto reductase family 1 member B10 (AKR1B10), and monoglyceride lipase (MGLL) were enriched in the most significant pathway, glycerolipid metabolism. Further, we found that low expression of ALDH2 is correlated with poor overall survival and oxaliplatin resistance. Finally, we found that combined treatment with ALDH2 inhibitor and oxaliplatin will reduce the sensitivity to oxaliplatin in p53 mutant HT29 cells. In conclusion, we demonstrate that ALDH2 may be a biomarker for oxaliplatin resistance status in CRC patients and bring new insight into treatment strategy for p53 mutant CRC patients.

## 1. Introduction

Colorectal cancer (CRC) is the most common cancer worldwide [1, 2]. Surgery followed by adjuvant chemotherapy with 5-fluorouracil, leucovorin, and oxaliplatin (FOLFOX) is the treatment of choice for nonmetastatic CRC [3, 4]. Oxaliplatin is the third-generation platinum-based antineoplastic agent [5], and approximately half of the oxaliplatin-treated patients develop oxaliplatin drug resistance [6, 7]. The underlying mechanisms of oxaliplatin resistance are multifactorial and poorly understood [8]. Decreased drug accumulation, enhanced tolerance to damage, elevated detoxification, alterations of pathways involved in cell cycle kinetics, and apoptosis inactivation are some of the proposed factors [8, 9].

p53 is the common tumor suppressor gene involved in cell cycle arrest, DNA repair, and apoptosis in cellular stress [10, 11]. p53 mutation is related to many types of cancer, and about 40 to 50% of sporadic CRC has p53 mutation which is involved in cell proliferation, migration, invasion, angiogenesis, and drug resistance of cancer [12, 13]. CRC with mutant p53 has been reported to be associated with oxaliplatin, irinotecan, 5-FU, and doxorubicin [14–16]. Oxaliplatin inhibits CRC cells by increasing and activating p53, whereas silencing p53 decreases the oxaliplatin effect by inducing drug efflux, disrupting cell cycle regulation, and evasion of apoptosis [17–19]. However, finding the specific target for oxaliplatin resistance in p53 mutant CRC patients needs to be studied.

Discovering a new target for oxaliplatin resistance in p53 CRC patients is of paramount importance in fighting against resistance. The emergence of DNA microarray technology in the past decade allows the concurrent assessment of thousands of genes [8, 20]. Gene expression profiling of human cancers has provided important insights into mechanisms and targets implicated in oncogenesis in several cancers [20, 21]. Chronic exposure to oxaliplatin induces different gene expression patterns in several CRC cells [22]. Although the response to oxaliplatin treatment is heterogeneous, studies of induced signaling pathways by prolonged oxaliplatin exposure in vitro revealed novel molecular targets for therapeutic interventions [22, 23]. Therefore, in our study, we used HT29 cells which carried mutated p53 and predicted possible target genes in oxaliplatin resistance by bioinformatics approaches, including constructing a protein-protein interaction network, predicting hub genes and pathways, and identifying gene expressions in tumor and normal tissues and overall survival. The findings may provide new insights into oxaliplatin resistance on CRC patients carried with mutant p53.

## 2. Materials and Methods

### 2.1. Microarray Data

The Gene Expression Omnibus (GEO) dataset GSE42387 (http://www.ncbi.nlm.nih.gov/geo/) generated by Jensen et al. was used [24]. The dataset was sequenced on the GPL16297 Agilent-014850 Whole Human Genome Microarray 4x44K G4112F (Agilent Systematic Name, collapsed probe version) platform. It comprises three parental human colon cancer cell lines of HCT116 (MSI, TP53 proficient, and K-Ras mutated), HT29 (MSS, p53 mutated, and K-Ras wild type), and LoVo (derived from a metastatic site; MSI, p53 proficient, and K-Ras mutated), of which the chemotherapy-resistant subsets are generated after 9 months of gradual exposure to increase concentrations of oxaliplatin or irinotecan. Gene expression profiles of parental and resistant cell lines cultured in a drug-free medium for 2~3 weeks were obtained and compared to identify gene expression changes associated with chemotherapy resistance. Differentially expressed genes between parental and resistant cell lines were recalculated with GEO2R online tool [25, 26]. The HT29 (MSS, p53 mutated, and K-Ras wild type) parental and oxaliplatin-resistant cell lines were selected because the HT29 cell line has p53 mutation and other two cell lines have proficient p53 [24]. The *p* values of the genes were calculated with the *t*-test method, and the Benjamini and Hochberg method was used to calculate adjusted *p* values (false discovery rate (FDR)) [27]. Genes with a log-fold change (FC) of 2 or -2, a *p* value of <0.05, and FDR < 0.05 were deemed to be DEGs. DEGs were plotted on a bidirectional hierarchical clustering heat map constructed using https://heatmapper.caand R package [27, 28].

### 2.2. Construction of the PPI Network

The STRING web tool (vers. 10.0, http://www.string-db.org/) was used to create the PPI networks [29]. The cutoff criterion is equal to 0.7 and greater than 0.7 score (high confidence). PPI pairs were input into Cytoscape software (vers. 3.7.0, http://www.cytoscape.org) and analyzed with the CytoNCA app for Cytoscape [30, 31]. Hub genes (highly connected genes) were determined by calculating the degree value (number of lines connecting the genes) with a cutoff of ≥2. The PPI network was created by using Cytoscape software and this online tool (http://genemania.org/).

### 2.3. Pathway Enrichment Analysis of DEGs

The DAVID web tool (https://david.ncifcrf.gov) was used to group hub genes from PPIs by their common pathways with reference to the Kyoto Encyclopedia of Genes and Genomes (KEGG, http://www.genome.jp/kegg/) database website with a Benjamini-Hochberg FDR value of <0.25 as a cutoff point [32]. Genes enriched in the pathways were plotted on a bidirectional hierarchical clustering heat map constructed using R package [19].

### 2.4. Clinical Validation of DEGs

DEGs involved in the pathways were clinically validated using UALCAN (TCGA database) and GENT2 (GSE database). Average expression levels of all of the target genes in normal vs. tumor tissues were represented by the UALCAN web tool. Expression levels of these genes were independently compared to the overall survival (OS) in months by the GENT2 web tool by plotting Kaplan-Meier survival curves. Hazard ratios (HRs) with 95% confidence intervals (CIs) and log rank *p* values less than 0.05 as the significance value were calculated.

### 2.5. Drug-Gene Interaction

The drug-gene interaction database (DGIdb) (https://dgidb.org/) was used to find the related drugs for the significantly enriched genes in the glycerolipid metabolism pathway.

### 2.6. Sulforhodamine B (SRB) Colorimetric Assay for Screening ALDH2 Inhibitor with Oxaliplatin Anticancer Effect in CRC Cells

The CRC cell lines, HT29, HCT116, and DLD-1, were purchased from American Type Culture Collection (ATCC) (Manassas, VA, USA). The cells were cultured in RPMI-1640 containing 10% fetal bovine serum (Gibco Life Technologies) and 1% penicillin–streptomycin (10,000 U/mL penicillin and 10 mg/mL streptomycin) at 37°C in 5% CO_2_ in a humidified incubator. Initially, 7 × 10^3^ cells (HT29 and HCT116) were seeded in each well of 96-well plates. After overnight incubation in the CO_2_ incubator, different doses of oxaliplatin (0~2.5 *μ*M) or vehicle with or without daidzein (0-100 *μ*M) were added into the wells and left for 48 h. Next, the treated cells were fixed with 10% trichloroacetic acid (Santa Cruz Biotechnology, CA, USA) overnight and then stained with protein-bound SRB for 30 min. After staining, cells were washed twice with 1% acetic acid to remove excess dye. A 10 mM Tris base solution was used to dissolve the protein-bound dye. The optical density was measured with a microplate reader at 515 nm (Bio-Rad Laboratories, Hercules, CA, USA).

## 3. Results

### 3.1. DEGs and PPI Networks

**Figure 1:**
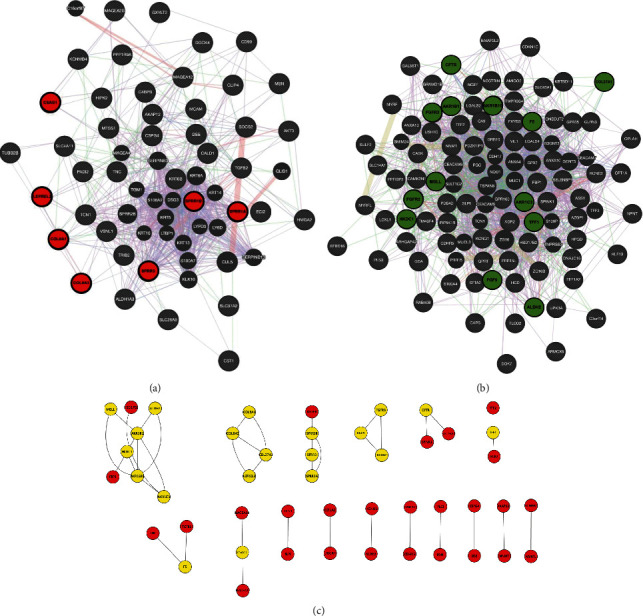
Protein-protein interaction (PPI) network of differentially expressed genes (DEGs). (a) Upregulated genes network and (b) downregulated genes network. PPI pairs constructed in STRING were imported into Cytoscape software as described in Methods and Materials. Red represents the upregulated network, while green represents the downregulated network. (c) The hub genes from Cytoscape software. The lines represent interaction relationships between nodes. The highlighted DEGs represent hub genes (degree ≥ 2). Cutoff for selecting hub genes is greater than or equal to 2 degrees.

In order to figure out the specific target for oxaliplatin resistance in p53 mutant CRC patients, the HT29 parental and oxaliplatin-resistant data (GSE42387 dataset) which consisted of 32,701 probe sets were applied for our study. 138 DEGs associated with HT29 parental and oxaliplatin-resistant cells were identified, and of which, 46 were upregulated and 92 were downregulated after GEO2R analysis. In order to investigate the biological significance of DEGs, the DEGs were screened and then loaded into the STRING database to get PPI pairs. These pairs were imported into Cytoscape software, and CytoNCA app was used to construct the PPI network and to identify the corresponding hub genes. The upregulated DEG network contained 41 nodes and 10 edges; and the degree ranged from 2 to 5 DEGs, including chondrosarcoma-associated gene 1 (CSAG1), collagen type IX alpha 3 (COL9A3), collagen type VIII alpha 1 (COL8A1), prolyl 3-hydroxylase 2 (LEPREL2), small proline-rich protein 1B (SPRR1B), small proline-rich protein 1A (SPRR1A), and small proline-rich protein 3 (SPRR3) that were identified as hub genes (Figures 1(a) and 1(c), Table 1). The downregulated network contained 86 nodes and 19 edges, and the degree ranged from 2 to 5.13 DEGs, including collagen type XXVII alpha 1 chain (COL27A1), hexokinase domain-containing protein 1 (HKDC1), aldo-keto reductase family 1 member C3, EC 1.(AKR1C3), cystic fibrosis transmembrane conductance regulator (CFTR), fibroblast growth factor 9 (FGF9), fibroblast growth factor receptor 2 (FGFR2), fibroblast growth factor receptor 3 (FGFR3), trefoil factor 1 (TFF1), coagulation factor 5 (F5), aldo-keto reductase family 1 member B1 (aldose reductase) (AKR1B1), aldo-keto reductase family 1 member B10 (AKR1B10), aldehyde dehydrogenase, mitochondrial, EC 1.2.1.3 (ALDH2), and monoacylglycerol lipase (MGLL) that were identified as hub genes (Figures 1(b) and 1(c), Table 1).

### 3.2. KEGG Pathway Analysis

**Figure 2:**
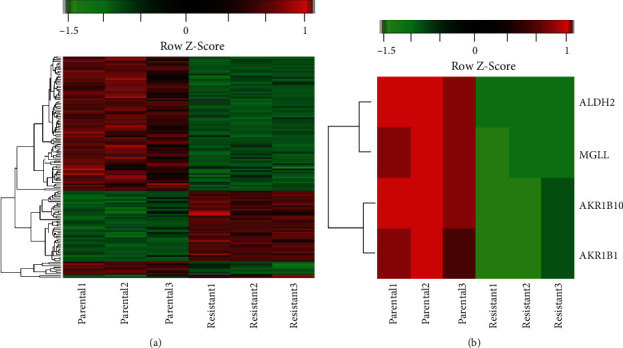
Heat map showing differentially expressed genes (DEGs) and genes enriched in the KEGG pathway. (a) A bidirectional hierarchical clustering heat map was constructed. Expression values are log-fold changes (>2 or <-2, with a false discovery rate of <0.05) between the corresponding oxaliplatin-resistant and the parental HT29 cells. (b) Significantly expressed genes in the glycerolipid metabolism pathway in oxaliplatin-resistant HT29 cells compared to parental HT29 cells. Black indicates no change in expression, green indicates downregulation, and red indicates upregulation, respectively.

The DAVID online tool was used to assort the gene functions of the 20 hub genes from the PPI networks using the KEGG reference. The genes including AKR1B1, AKR1B10, HKDC1, ALDH2, and MGLL were enriched in the glycerolipid, galactose, fructose, and mannose metabolism pathways. The KEGG pathway analysis indicated that one pathway, the glycerolipid metabolism pathway, reached statistical significance (*p* < 0.05, FDR (false discovery rate) < 0.25) for the downregulated group. The downregulated genes, AKR1B1 AKR1B10, ALDH2, and MGLL, were enriched in the glycerolipid metabolism pathway which is critical for the balance of lipid storage and construction of the membrane of cells (Table 2). The hub genes enriched in the glycerolipid metabolism pathway by a bidirectional hierarchical clustering analysis were listed, and the expression of DEGs was presented (Figures 2(a) and 2(b)).

### 3.3. Clinical Validation of DEGs

**Figure 3:**
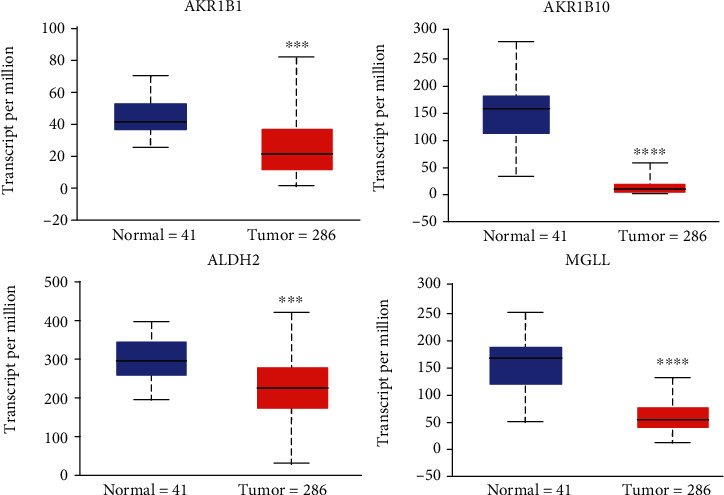
Box plots show the expression of significant genes in normal and tumor samples. The TCGA database showed the expression level of AKR1B1, AKR1B10, ALDH2, and MGLL in normal and tumor samples of colorectal cancer. Averages of specific differentially expressed gene (DEG) expression levels were dichotomized by the median value. ^∗∗∗^*p* < 0.001 and ^∗∗∗∗^*p* < 0.0001.

**Figure 4:**
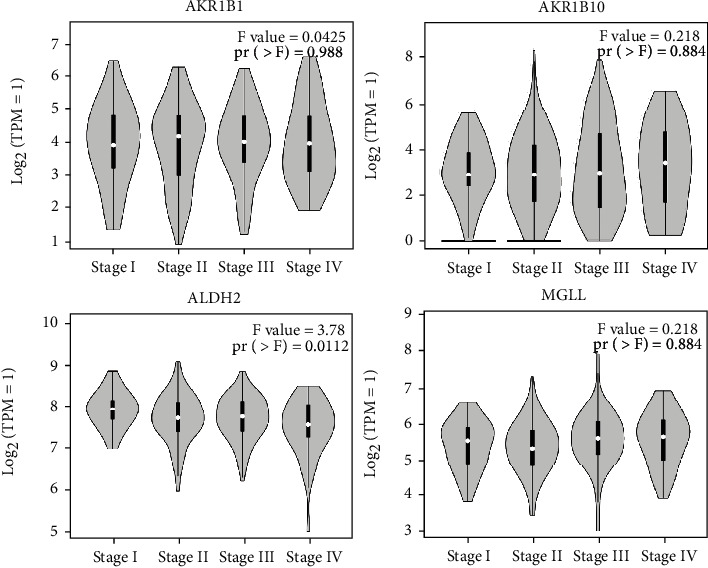
Expression level of significant genes in different stages of CRC. The GEPIA online tool was used to determine the expression of ALDH2, AKR1B1, AKR1B10, and MGLL genes in 4 stages of CRC. *p* < 0.05 was considered statistically significant.

**Figure 5:**
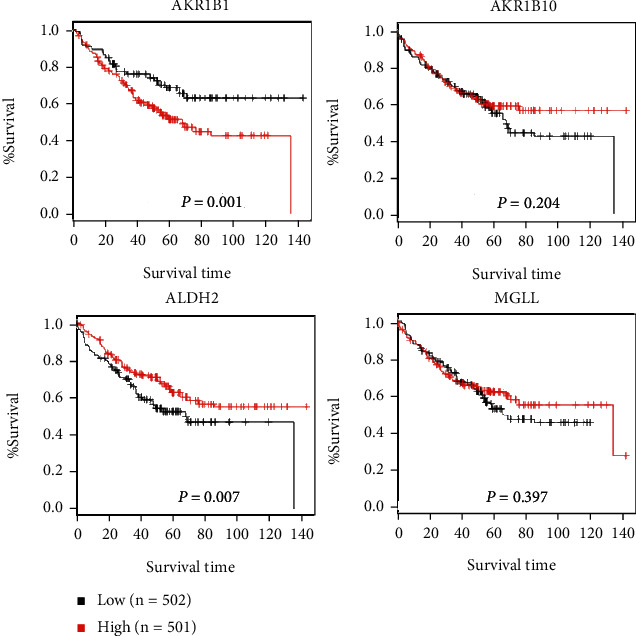
Kaplan-Meier survival curves present prognostic relationship of gene expression and overall survival of specific genes. High expression of ALDH2, AKR1B10, and MGLL was related to better outcome, while high expression of AKR1B1 was related with poor overall survival.

To further check the clinical roles of the DEGs enriched in the KEGG pathways, their associations with CRC patient survival were assessed. The UALCAN web tools were used to analyze the AKR1B10, AKR1B1, MGLL, and ALDH2 genes in the COAD and CRC dataset. The UALCAN database showed that the genes were significantly downregulated in COAD (colon adenocarcinoma) tissue compared to the normal tissue using 41 normal tissues and 286 tumor tissues (*p* < 0.001) (Figure 3). Expression of genes in different stages was determined by GEPIA online tool. The expression of ALDH2 was significantly decreased when the stages get higher (Figure 4). The correlation of significant genes to survival curves was plotted using the GENT2 online tool. The results are presented visually by Kaplan-Meier survival plots. *p* < 0.05 was considered statistically significant. GENT2 online tools also showed that the low expression of AKR1B1 is associated with good overall survival (OS) (*p* = 0.001), while the low expression of ALDH2 was related to poor overall survival using a total of 1003 patients (*p* = 0.007). However, the expression of AKR1B10 (*p* = 0.204) and MGLL (*p* = 0.397) is not related to the overall survival of CRC patients (Figure 5).

### 3.4. Drug-Gene Interaction

Interaction of ALDH2, AKR1B1, AKR1B10, and MGLL genes with drugs was determined by the drug-gene interaction database (DGIdb). We found prunetin, guanidine, and disulfiram as inhibitors for ALDH2. Disulfiram is an inhibitor of ALDH2 with an interaction score of 0.94. Disulfiram is an approved drug for the first-line therapy to treat alcoholism [33]. Guanidine is a small molecule that functions as an acetylcholine-releasing agent and is an FDA-approved drug for reducing symptoms of muscle weakness and easy fatigability caused by myasthenic syndrome of Eaton-Lambert [33, 34]. However, DGIdb did not show that guanidine's information about direct interaction with ALDH2 was not available and its interaction score was 7.65. We found several inhibitors, lidorestat, fidarestat, zopolrestat, tolrestat, zenarestat, sorbinil, exisulind, and sulindac, for AKR1B1 while fidarestat, exisulind, and sulindac for AKR1B10. Among them, sulindac is an FDA-approved nonsteroidal anti-inflammatory drug (NSAID) for inhibiting COX enzyme, with an interaction score of 0.79. For the MGLL gene, DGIdb did not show any interacted drugs (Table 3).

### 3.5. ALDH2 Inhibitor Increases the Resistance to Oxaliplatin Treatment

**Figure 6:**
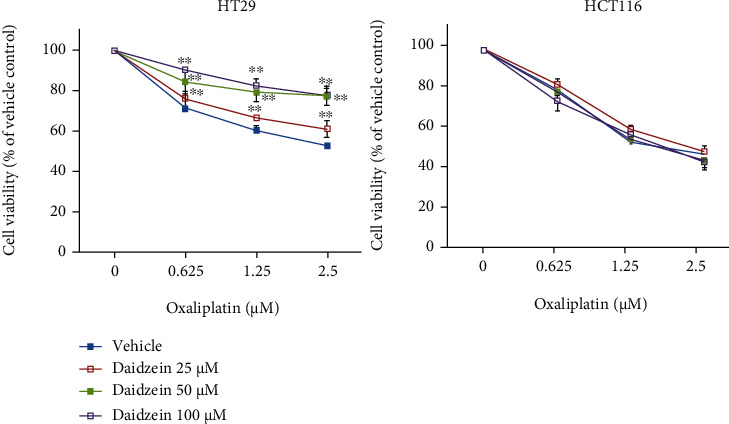
The effect of ALDH2 inhibitor in the sensitivity to oxaliplatin on CRC cells. HT29 (p53mutant) and HCT116 (p53 wild type) cells were incubated with different doses of oxaliplatin (0-2.5 *μ*M) with different doses of daidzein (0-100 *μ*M). The cell viability was determined by SRB assay. In HT29 cells, cotreatment with daidzein will decrease the sensitivity to oxaliplatin treatment. There is no difference in HCT116 cells. All the experiments were performed at least three times independent. ^∗∗^*p* value < 0.01.

To confirm the role of ALDH2 in oxaliplatin therapeutic efficacy in CRC, we cotreated with ALDH2 inhibitor (daidzein) with oxaliplatin in HT29 and HCT116 cells. As shown in Figure 6, HT29 and HCT116 were incubated with different doses of (0-2.5 *μ*M) oxaliplatin, and the cell viability was determined by SRB. The cell viability was negatively correlated to the amount of oxaliplatin. HT29 is more resistant to oxaliplatin than HCT116. Further, cotreated with different doses of daidzein (ALDH2 inhibitor) in HT29 and HCT116 cells, we found that cotreatment with daidzein increases significantly cell viability in HT29, but there is no influence in HCT-116 cells. Those results indicate that suppression of ALDH2 activity will influence the oxaliplatin sensitivity in p53 mutant HT29 cells.

## 4. Discussion

The HT29 cell line is the p53 mutant CRC cell line, and it is known that resistance to oxaliplatin treatment is associated with p53 mutant or loss of p53 [14–19]. Finding the potential target genes in p53 mutant patients with oxaliplatin resistance is important in the clinical practice. The combinatorial usage of the cytotoxic drug, oxaliplatin, with 5-fluorouracil plus leucovorin (FOLFOX) with/without biological agents as first-line therapy for metastatic CRC, has been proven to have a response rate of >50% and has prolonged overall survival [35]. Despite improvements in treatments for metastatic CRC, both inherited and acquired resistance to oxaliplatin is still a major cause for therapeutic failure. Resistance to oxaliplatin in CRC contributes to low OS rates despite recent advances in medical and surgical therapies [5, 32]. Identifying molecular targets which can predict disease progression and therapeutic responses will help in improving survival times and the quality of life of CRC patients [5]. In our study, DEGs regarding oxaliplatin resistance were obtained from GEO2R analysis, and after KEGG analysis, 4 of the downregulated hub genes were found to be enriched in glycerolipid metabolism with degree ≥ 2. Cancer cells regulate the activation of lipid anabolic metabolism, which involves the process of lipid synthesis, storage, and degradation; related signaling networks for membrane formation and energy storage; and production of signaling molecules and works as a vital energy source to generate ATP via fatty acid oxidation (FAO) under conditions with low energy [34].

ALDH2 is a gene that plays an essential regulatory role in alcohol metabolism and was also reported important in initiation of cancer progression of CRC and pancreatic cancer [33, 36–38]. ALDH2's polymorphism was associated with risk of CRC [33, 36–38]. More studies reported that different polymorphisms of ALDH2 in various populations are associated with the risk of CRC and rs1329149 T/T genotypes were associated with increased risk of getting CRC in southwestern Chinese population [39–43]. In addition, ALDH2 is required for the conversion of retinol (vitamin A) to retinoic acids [44]. The chemopreventive and therapeutic characteristics of natural retinoids including ATRA, 9-*cis*RA, and 13-*cis*RA, against colorectal cancer, have been investigated [45]. It was reported that ATRA repressed invasiveness of CRC cells by downregulating matrilysin [46]. Natural retinoids such as all-trans-retinoic acid inhibit colon cancer HT29 cells by decreasing COX-2 and C/EBP expressions [47]. Also, 9-cis-retinoic acid suppressed cancer cells in CRC by increasing apoptosis and inhibiting COX-2 through the activation of PPAR*γ* [48], while retinal suppressed CRC by inducing HOXA5 and repressing stem cell markers prominin 1 and ALDH1 [49]. A previous study showed that ADH2^∗^1^∗^2 and the p53 codon 72 Pro/Pro genotypes increased the risk of esophageal SCC [50]. Thus, tumor with low expression of ALDH2 cannot convert retinol to retinoic acid, resulting tumor progression. Another study assessed the several isoforms of the ALDH family such as ALDH1A3, ALDH3B1, ALDH2, and ALDH7A1 to be related with survival in HPV16+/p53WT head neck squamous cell cancer. They reported that rep53 functional states are associated with distinct ALDH isoforms [51].

Also, it is known that there is an inhibitor of ALDH2, disulfiram, which was used as the first-line therapy to treat alcoholism [52]. Our result showed that the resistant cell line of CRC has low expression of ALDH2, and low expression of ALDH2 is also associated with poor overall survival of CRC. Furthermore, cotreatment with daidzein (ALDH2 inhibitor) caused an increase in cell viability in oxaliplatin-treated HT29 cells, but there is no influence in HT116 cell (Figure 6). Therefore, based on our findings, using an ALDH2 inhibitor such as disulfiram in oxaliplatin-treated CRC patients should be avoided to prevent unnecessary drug resistance. HT29 is the p53 and APC mutant cell line. APC mutation was not associated with the responses to oxaliplatin-based and irinotecan-based chemotherapy [53]. However, APC mutation in the HT29 cell line may affect the response for oxaliplatin treatment in some way. Chang et al. reported that treating the patients with KRAS mutant tumors with oxaliplatin had better overall survival than irinotecan even though it was not statistically significant [53]. Thus, a clinical study regarding this issue also needs to be done in the future.

The aldo-keto reductases (AKRs), AKR1B1 and AKR1B10, are involved in reductive metabolism of endogenous signaling molecules and the detoxification of xenobiotics and are known oncogenes with different expression levels in different tumors [54]. These genes are said to be prognostic and potential drug targets [55]. AKR1B1 is an aldo-keto reductase (AKR), which plays a role in cellular defense and signaling, and is involved in complications of diabetes and tumor progression in basal-like breast cancer (BLBC), CRC, lung cancer, and pancreatic cancer [56–62]. AKR1B1 was also considered as target gene for vincristine in CRC and a screening marker of CRC [58, 63]. The FDA-approved drug sulindac which works as an inhibitor of AKR1B1 was found by the drug-gene interaction database. Sulindac is a NSAID which inhibits cyclooxygenase (COX) enzyme and is reported to have chemoprevention activity for adenomatous colorectal polyps and colon cancer [64, 65]. Furthermore, usage of NSAIDs such as indomethacin inhibits the growth of colon tumor in vitro and in vivo [66, 67]. Sulindac and its metabolites suppressed the proliferation of RKO and SW480 colon cancer cells [68]. Therefore, using the NSAID might be related to oxaliplatin resistance of colorectal cancer.

Our result showed that AKR1B1 was downregulated in colon tumor and in oxaliplatin-resistant cell lines. However, the GENT2 database showed that its high expression is related to poor overall survival. These conflicting results may be because of different stages of CRC samples or limited number of samples. Therefore, further study is needed for solving this issue. AKR1B10 is reported to be related with chemoresistance of several cancers [69–71]. The decreased level of AKR1B1 was reported to be involved in small percent of colorectal cancer, whereas decreased expression of AKR1B10 was found in most of colorectal cancer samples [72]. It is known that AKR1B10 was upregulated in wild-type p53 cancer cells, while it was downregulated in mutant p53 cancer cells [73]. Our result also showed that colon tumor has a low expression of AKR1B10 and patients with higher expression of AKR1B10 have better overall survival. So far, no drugs for treating low expression of AKR1B10 have been found. Therefore, investigations for AKR1B10 being a potential target for CRC are in need.

Deficiency of MGLL (monoacylglycerol lipase) is involved in oral carcinoma, metastatic hepatocellular carcinoma, breast cancer, and GIST [74]. In addition, the low expression of MGLL is related with poor overall survival of HCC [75] and considered a tumor suppressor in HCC [54]. Deficiency of *MGLL* is also associated with colorectal cancer progression along with other genes by regulating ECM and metabolism [76–78]. MGLL is characterized as a direct PRDM5 target in human colon cancer cells and in *Prdm5* mutant mouse intestines [79]. *PRDM5* is a tumor suppressor gene, and loss of this gene is related with colon cancer tumorigenesis [80]. It is reported that glycerolipid metabolism plays an essential role in colorectal polyp which highly increases the risk of CRC [81].

The present study has some limitations, and further studies using mouse models would be helpful. Oxaliplatin resistance in CRC can also be affected by many factors in addition to the abovementioned genes because of complexity of tumor biology including tumor microenvironment, stem cell niches, and microbiota. This study discovered that interactions among multiple genes in different pathways were responsible for oxaliplatin resistance for p53 mutant CRC patients. We have demonstrated a possible target in CRC that can improve the therapeutic index of oxaliplatin, suggesting that unnecessary drugs should be avoided for patients with oxaliplatin-resistant CRC. Therefore, the detailed mechanism of ALDH2 in oxaliplatin resistance of p53 mutant CRC needs to be investigated in further studies.

## Figures and Tables

**Table 1:** Hub genes in the upregulated or downregulated PPI network.

Gene ID	Gene name	Degree
*Hub genes in the upregulated PPI network*		
*SPRR1B*	Small proline-rich protein 1B	3.0
*SPRR1A*	Small proline-rich protein 1A	2.0
*SPRR3*	Small proline-rich protein 3	2.0
*COL8A1*	Collagen type VIII alpha 1	2.0
*LEPREL2*	Prolyl 3-hydroxylase 2	2.0
*COL9A3*	Collagen type IX alpha 3	2.0
*CSAG1*	Chondrosarcoma-associated gene 1	2.0
*Hub genes in the downregulated PPI network*		
*AKR1B1*	Aldo-keto reductase family 1 member B1 (aldose reductase)	5.0
*AKR1B10*	Aldo-keto reductase family 1 member B10 (aldose reductase)	5.0
*AKR1C3*	Aldo-keto reductase family 1 member C3, EC 1.	3.0
*HKDC1*	Hexokinase domain-containing protein 1	3.0
*COL27A1*	Collagen type XXVII alpha 1 chain	3.0
*ALDH2*	Aldehyde dehydrogenase, mitochondrial, EC 1.2.1.3	2.0
*MGLL*	Monoacylglycerol lipase	2.0
*FGFR3*	Fibroblast growth factor receptor 3	2.0
*FGF9*	Fibroblast growth factor 9	2.0
*FGFR2*	Fibroblast growth factor receptor 2	2.0
*TFF1*	Trefoil factor 1	2.0
*F5*	Coagulation factor 5	2.0
*CFTR*	Cystic fibrosis transmembrane conductance regulator	2.0

**Table 2:** Enriched KEGG pathways.

Term	FDR	*p* value	Genes
^∗^Glycerolipid metabolism	0.006285	0.0001	AKR1B10, AKR1B1, ALDH2, MGLL
Galactose metabolism	0.023746	0.0011	AKR1B10, AKR1B1,HKDC1
Fructose and mannose metabolism	0.023746	0.0013	AKR1B1, AKR1B10, HKDC1

KEGG: Kyoto Encyclopedia of Genes and Genome. ^∗^FDR (false discovery rate) < 0.25.

**Table 3:** Interaction of significant genes with drugs by the drug-gene interaction database.

Drug name	Prunetin	Guanidine	Disulfiram	Lidorestat	Zenarestat	Zopolrestat	Tolrestat	Fidarestat	Sorbinil	Sulindac	Exisulind
AKR1B1				IS:8.74	IS:4.37	IS:3.28	IS:2.91	IS:3.28	IS:2.19	IS:∗0.79	IS:0.49
AKR1B10								IS:7.65		IS:0.7	IS: 1.7
ALDH2	IS:12.24	IS:^∗^7.65	IS:^∗^0.94								
MGLL											

^∗^FDA-approved drug clinically available. IS: interaction score.

## Data Availability

All the underlying data supporting the results of our study are in the manuscript.
